# Community Engagement Within the Evaluation of Public Policies for Zoonotic Spillover Prevention: A Secondary Matrix Analysis

**DOI:** 10.3390/ijerph22050797

**Published:** 2025-05-18

**Authors:** Nicole Redvers, Yasaman Mohammadi Kamalabadi, Danya Carroll, Mohammad Yasir Essar, Omnia El Omrani

**Affiliations:** 1 Schulich School of Medicine and Dentistry, University of Western Ontario, London, ON N6G 2M1, Canada; ymoham24@uwo.ca (Y.M.K.); dcarro4@uwo.ca (D.C.); messar@uwo.ca (M.Y.E.); 2 The Blavatnik School of Government, University of Oxford, Oxford OX2 6GG, UK; omniaelomrani0@gmail.com

**Keywords:** community engagement, equity, spillover, pandemic prevention, zoonotic spillover prevention, public policies, evaluation, content analysis, community consultation, relational community engagement, biosecurity, community-based research, CBPR, community consultation

## Abstract

Despite the overall health, economic, and social costs of zoonotic spillover, its impacts are not felt equally around the globe. Engaging local communities in primary spillover prevention may help to better ensure equity is considered in research and policy-making activities. Our study aimed to gain an understanding of how and at what level community engagement (CE) has been incorporated into the evaluation of public policies for zoonotic spillover prevention. We conducted a secondary analysis on an existing dataset from a systematic review, beginning with a structured deductive content analysis. A secondary matrix of analysis was engaged using an adapted CE tool for screening the included articles based on their level of CE. We then characterized relevant themes based on the CE elements within the included articles. Of the 95 articles included, 55 had no level of CE reported. Among the included articles that had some level of CE, elements included the platforming of community consultation, community training for involvement in spillover prevention research, cultural and language considerations being engaged, community protection and awareness programmes for public health and biosecurity, and community-centered data collection processes being engaged. Our findings highlight the persistent equity gaps in appropriately engaging affected communities within the evaluation of public policies for spillover prevention.

## 1. Introduction

The majority of emerging infectious diseases have been transmitted between animals and humans (i.e., spillover) [1]. Spillovers have precipitated five viral pandemics affecting humans (1918 influenza, 1957–1958 influenza, 1968 influenza, 2009 influenza, and HIV) since 1918, with additional evidence pointing to COVID-19 as the result of spillover [2,3,4,5,6,7,8]. With the H5N1 bird flu recently causing outbreaks in poultry and United States (US) dairy cows (with several human infections confirmed) [9,10], there is continued concern about the lack of primary spillover prevention activities occurring around the globe, with the main focus being on post-spillover containment efforts [11]. Past viral pandemics stemming from spillover have had exorbitant health costs as well as economic, social, and environmental costs [12,13,14]. Without greater attention to primary spillover prevention in the context of a changing world, the risks and costs to humans, animals, and the environment will continue to be amplified [15].

Despite the overall health, economic, and social costs of viral spillover, it is known that these potential or ongoing costs are not felt equally around the globe [16]. Low- and middle-income countries, as well as people of lower socioeconomic status living in high-income countries, bear a disproportionate burden from the effects of pandemics [17]. Despite these disproportionate impacts, “equity-centered planning, decision-making and action”, as well as the “attentiveness to power and the relationship between political economy and health”, have often been minimized in global pandemic governance [18]. The evolving World Health Organization (WHO) pandemic treaty work has spurred increasing calls for equity considerations to play a central role [19]. Equity considerations, however, have often been with respect to “post-spillover secondary pandemic prevention, which focuses on actions taken after a pathogen has spilled over from animals to humans” and which is often referred to as preparedness and response [20,21]. Primary pandemic prevention (i.e., “actions taken to reduce the risk of an outbreak occurring at all”) has received no explicit mention in the draft texts [20], which have an ongoing focus on downstream interventions relevant after a pathogen is circulating in humans [21]. Thus, it is hard to grasp how equity will be fully engaged and operationalized in pandemic prevention efforts going forward.

One of the key barriers to appropriate equity incorporation into primary spillover research and policy development activities is the lack of available information on equity, as well as outcomes related to equity. Barriers also include limited access to relevant data and the differences in healthcare infrastructure and disease surveillance systems that disproportionately affect low-income countries, further undermining equity across varied geopolitical realities. Additionally, there has been noted concern from rights-holding groups, including Indigenous Peoples, that potential spillover prevention activities may further strip them of their enshrined rights to their lands and cultural practices [22]. Although equity is often assumed within some frameworks used within primary pandemic prevention activities (e.g., One Health), evidence has demonstrated that research has not been very accommodating to other worldviews or paradigms within related work [23]. For example, within a systematic review carried out in 2021, it was found that there was no significant connection between One Health and Indigenous knowledge established in the analyzed articles, and any implications from utilizing One Health with regard to Indigenous Peoples and culture were not explicitly addressed [23]. With this, more research is needed to understand how equity is being considered and integrated in primary spillover prevention-related activities, including research, policy, and practice.

Relevant local community involvement in primary spillover prevention may help ensure that equity considerations are integrated into pandemic prevention research and practice. Meaningful community engagement can also serve as a benchmark for assessing the level of collaboration in partnerships with affected communities [24]. With this, our study aimed to gain a clearer understanding of how and at what level community engagement has been incorporated into the evaluation of public policies for zoonotic spillover prevention. We developed a protocol to carry out a secondary matrix analysis on an already published systematic scoping review [25], with the specific overall goal of assessing the level of community engagement in the included research using an adapted community engagement tool.

## 2. Materials and Methods

### Research Question and Study Design

Our research question for this work was as follows: What level of community engagement has been used in the evaluation of public policies for zoonotic spillover prevention? For the purposes of this study, community engagement is defined, as per the World Health Organization (WHO), as a “process of developing and maintaining relationships that enable people to work together to address health-related issues and promote well-being to achieve positive and sustainable health impact and outcomes” [26]. Additionally, the framing of “community” in this study is viewed as being a group of people with a shared interest or concern and who are directly affected by any policy in question.

This study is a further analysis of a systematic scoping review carried out by a multi-country research team led from Canada [25]. The original review by Astbury et al. aimed to identify and describe evaluations of public policies that targeted the determinants of zoonotic spillover [25]. The research team in the original paper searched “Medline, SCOPUS, Web of Science and Global Health in May 2021 using search terms combining animal health and the animal-human interface, public policy, prevention and zoonoses”, and also searched relevant organizations’ websites for evaluations published in the grey literature; “all evaluations of public policies aiming to prevent zoonotic spillover events were eligible for inclusion” [25]. The original review identified 95 relevant publications evaluating 111 policies with 27 unique policy options, including, as examples, habitat protection trade regulations, border control and quarantine procedures, farm and market biosecurity measures, public information campaigns, and vaccination programmes, as well as multi-component programmes [25].

For our current study, we first carried out structured deductive content analysis, as described by Kyngäs & Kaakinen [27], on the 95 papers included in the review by Astbury et al. [25]. Structured deductive content analysis allows space for working with conceptual material already developed while seeking to assess or apply it in another context [27]. Given this, we were then able to conduct a secondary matrix of analysis using a community engagement tool that we adapted from Vaughn & Jacquez [28] (i.e., participation choice points in the research process diagram), and Key et al. [29] (i.e., community-engaged research (CEnR) diagram), to further analyze the relevant body of literature [30]. Our developed tool merged the headings and descriptions from Vaughn & Jacquez [28] and Key et al.’s [29] respective figures to ensure a well-rounded analysis of community engagement in the included papers. We subsequently outlined six potential levels of community engagement that a paper could be graded with (i.e., our matrix), which are listed in Table 1. Using structured deductive content analysis as previously noted [27], each paper was given only one numerical score depending on the level of community engagement that was explicitly noted within the included papers (i.e., our analysis). Less or no community engagement was given a lower score than higher levels of community engagement (see Table 1).

The structured deductive content analysis was conducted by two independent reviewers (YMK, MYE). In cases of discrepancies in the analysis matrix, an additional reviewer (DC, NR) facilitated a group discussion to reach a resolution. A Microsoft Excel 365 ProPlus spreadsheet was used to track articles containing evidence of community engagement in research activities. Articles with some level of community engagement were assigned a numerical score from 1 to 5 (see Table 1), while a score of 0 was given if none was noted. For scores from 1 to 5, direct evidence from the paper justifying the rating was recorded in the spreadsheet. Qualitative content analysis, following Elo & Kyngäs [31], was then applied to the collected evidence to identify key community engagement elements in the articles reviewed by Astbury et al. [25]. The detailed findings are reported below.

## 3. Results

The general characteristics of the articles included in the original review were previously reported in detail by Astbury et al. [25]. As noted previously, this original review identified 95 relevant publications evaluating 111 policies with 27 unique policy options, including habitat protection trade regulations, border control and quarantine procedures, farm and market biosecurity measures, public information campaigns, and vaccination programmes, as well as multi-component programmes [25]. In our secondary analysis of the included articles on their reported level of community engagement in the evaluation of public policies for zoonotic spillover prevention, the majority (*n* = 55, 57.9%) of the articles received a score of zero, indicating no evidence of community involvement (see Table 1 and Table 2). Scores of one and two were assigned to 12.6% (*n* = 12) and 24.2% (*n* = 23) of the included articles, respectively. A smaller proportion achieved higher scores, with 3.1% (*n* = 3) receiving a community engagement score of three, and 2.1% (*n* = 2) a score of four. Notably, no article achieved the highest score of five, which reflected community-driven or -led engagement (see Table 1 and Table 2).

For included articles which scored at least a level one on the community engagement scale, there was a diverse range of community members and stakeholders engaged, including consumers (e.g., patients [100], purchasers of live poultry products [113]), distributors and suppliers (e.g., live bird markets operators [99], vendors [103], traders [108], pet feed shops [91], fishermen [112], and horse industry groups [111]), producers (e.g., farmers [88,101], pig rearers [109], poultry raisers [98,120,123]), experts and specialists (e.g., experts on animal brucellosis [117], pharmacist [122], veterinary professionals [96,114,122]), and leaders (e.g., market managers [125], animal health officers [92], community leaders [123], directors of the animal health department [103], and village or tribal chiefs [101,122]). These diverse community members played various roles. Some responded to questionnaires and surveys or were engaged in educational programmes aimed at raising awareness about spillover prevention. Some additionally became trainees actively involved in the research process, while others served as informants or experts who were consulted for their knowledge and experience.

Content analysis was ultimately carried out on the forty articles that had a community engagement score from 1 to 4 (no papers had a score of 5). Five main themes were characterized in these papers, which are outlined in Table 3 and described further below. It is important to note, however, that, out of these forty papers, thirty-five had a community engagement score of only 1 or 2. Given this, the themes outlined most accurately reflect community-informed, community consultation, and community-involved approaches, as opposed to community collaborative or community-driven approaches.

### 3.1. Platformed Community Consultation in Spillover Prevention Research

Community experts were consulted in a meaningful way to ensure that research methods and health interventions were contextually appropriate, culturally relevant, and aligned with community needs. For example, Samaan et al. [125] used a participatory approach by organizing monthly consultation meetings with market managers, sanitation teams, and poultry vendors. These sessions provided a platform to discuss challenges, propose solutions, and collaboratively implement infrastructural and behavioral interventions [125]. Through continuous dialogue, community insights informed practical strategies, making it possible to achieve compliance with key measures not previously practiced in the markets [125]. Similarly, Thomas et al. [126] highlighted the importance of incorporating feedback from community stakeholders in the development of a questionnaire. Working with the director of the rabies control programme, the research team identified salient beliefs and concerns raised by dog owners over five years [126]. The draft questionnaire was piloted with six dog owners across three villages to ensure it was comprehensive, clear, and applicable [126]. Feedback from these community experts led to refinements that enhanced the instrument’s usability and relevance [126]. Turkson et al. [120] further demonstrated the value of consulting with stakeholders by designing questionnaires that explored socioeconomic factors influencing the adoption of preventive and containment measures. Open-ended questions allowed the stakeholders to provide detailed explanations of their perspectives, complementing quantitative data collected through Likert-scale items [120]. Overall, this theme highlights how consulting with community experts can strengthen research tools and intervention strategies by integrating specialized, context-specific knowledge from relevant communities.

### 3.2. Community Training for Involvement in Spillover Prevention Research

Efforts were made to actively involve community members and local stakeholders in the process. This included providing education and training that would equip them to participate in the planning, implementation, and evaluation phases of health interventions and research activities. For example, Bechir et al. [122] showed the importance of involving representatives of nomadic pastoralists in vaccination campaigns. The nomadic pastoralists contributed a unique understanding of seasonal movements that enabled successful outreach to remote groups [122]. This collaboration ensured that vaccination services reached populations that are often underserved [122]. Similarly, Hunter et al. [123] emphasized the importance of training local individuals. Indonesian anthropology graduates were trained to support research activities, such as facilitating focus groups, conducting interviews, and transcribing data [123]. Their involvement was key to interpreting community attitudes, behaviors, and traditional poultry-raising practices to improve avian influenza prevention. This approach not only built local capacity but also ensured culturally informed and contextually relevant data collection [123]. Oladokun et al. [116] further emphasized the role of community training in health interventions. Poultry farmers and rural flock holders were trained to recognize and report suspected cases of avian influenza [116]. This engagement strengthened passive surveillance systems and improved disease control through partnerships with veterinary authorities, demonstrating how knowledge transfer can enhance early detection efforts [116]. Likewise, Kung et al. [111] showed effective engagement by involving horse industry groups in disseminating surveys to stakeholders. These groups actively supported research efforts by sharing survey URLs on their official websites [111]. These examples highlight the importance of tailored education and training programmes in fostering community ownership and improving implementation outcomes.

### 3.3. Cultural and Language Considerations Engaged Within Spillover Prevention Research Activities

Cultural and linguistic considerations were accommodated to ensure effective communication and data collection in spillover prevention research. For example, Bechir et al. [122] tailored health messages to nomadic pastoralists by organizing discussions in the culturally relevant language and using locally created images to support message delivery. Similarly, Bonwitt et al. [101] conducted semi-structured interviews in English, Mende, or Krio, allowing participants to respond in their preferred language. This language flexibility fostered deeper discussions about public health messages and practices related to wild meat consumption [101]. Brooks-Moizer et al. [103] and Guerrier et al. [109] further emphasized cultural responsiveness by utilizing translators and conducting interviews in languages spoken by participants, such as Vietnamese, Wallisian, Futunian, or French. Hunter et al. [123] additionally demonstrated a comprehensive approach by training local researchers to conduct focus groups and interviews in Balinese, Sasak, or Indonesian. This better ensured that data collection reflected participants’ cultural and linguistic contexts. These efforts highlight the importance of adapting research tools to honor cultural diversity and improve participant engagement.

### 3.4. Community Protection and Awareness Programmes for Public Health and Biosecurity

A range of integrated initiatives was introduced aimed at enhancing biosecurity measures, safeguarding animal health, and promoting community well-being. These efforts combined education, awareness-raising, and protective strategies to build resilience and prevent disease transmission. Programmes included training sessions [125], mass awareness campaigns [116], reporting systems [99], and targeted biosecurity interventions [87]. For instance, Oladokun et al. [116] reported the use of workshops, seminars, and media campaigns to educate farmers on biosecurity practices. Samaan et al. [125] emphasized sustainability by conducting tailored education sessions for market vendors and sanitation teams focusing on hygiene practices, waste management, and avian influenza detection. Bechir et al. [122] emphasized the importance of culturally sensitive approaches, using locally relevant discussions, nomadic-origin artists, images, and health messaging to effectively convey the importance of vaccinating children and women. Additionally, Vivancos et al. [98] increased vaccination awareness among poultry workers by distributing Department of Health information leaflets. This leaflet distribution process highlighted the programme’s goal of preventing genetic reassortment and clarified that seasonal influenza vaccines do not protect against avian influenza A [98]. In addition to education and training, a variety of protective systems and measures were implemented to strengthen biosecurity and public health. For example, the study by Akunzule et al. [99] detailed the establishment of regional communication hotlines. These hotlines enabled rapid public reporting of suspected Highly Pathogenic Avian Influenza (HPAI) cases for prompt intervention [99]. They also trained farmers on biosecurity measures, such as isolating newly sick animals; restricting movement of animals, people, and equipment; cleaning/disinfection procedures; and keeping poultry in closed housing to prevent exposure to wild birds. Andronico et al. [87] described stringent measures such as movement restrictions, surveillance, and enforced biosecurity within designated zones around infection points. These measures complemented pre-emptive culling and hunting bans to limit avian influenza H5N8 spread [87]. This section highlighted the value of introducing a broad range of measures to improve biosecurity and reduce the spread of viruses.

### 3.5. Community-Centered Data Collection for Spillover Prevention Research

Community engagement in the studies was enhanced through the use of diverse data collection strategies, such as interviews, group discussions, and direct observation. Informed consent was a critical component, as emphasized by Ferguson et al. [107], who highlighted its role in gaining trust and active participation. Brooks et al. [103] additionally demonstrated participant self-recruitment, showing how individuals willingly engaged in studies to support data collection. For more inclusive sampling processes, Lin et al. [113] utilized a combination of field-based and online recruitment methods to sample live poultry consumers across fifteen cities. Their process illustrated the importance of adapting recruitment strategies to target diverse populations [113]. Similarly, Manyweather et al. [114] explored self-selection (i.e., voluntary choice to participate) among horse owners living near previous Hendra virus cases to investigate vaccine hesitancy in a highly relevant subgroup.

Employing various data collection methods helped to ensure comprehensive and reliable datasets. Amparo et al. [100] conducted systematic and random household interviews to gather information on household size and animal bite incidence. Andronico et al. [87] integrated surveillance data including farm production types, infection timelines, and movement restrictions to analyze disease dynamics. Brinkley et al. [102] adopted a triangulated approach, creating databases of animal shelters and conducting telephone interviews with directors to examine trends in chicken intake.

Culturally embedded approaches further enhanced the degree of engagement and quality of data collection. For example, Guerrier et al. [109] combined pretested questionnaires with qualitative interviews involving key community figures to explore local knowledge of brucellosis among pig farmers. Similarly, Hunter et al. [123] conducted in-depth interviews with community members from affected and unaffected regions to compare knowledge, attitudes, and responses to HPAI outbreaks. Brooks et al. [103] utilized semi-structured interviews with bird vendors in Hanoi to assess trade practices, legislation enforcement, and vendor demographics, tailoring their methods to reflect local contexts and practices.

Direct observation complemented other data collection methods by providing validation and identifying environmental risk factors for spillover. Akunzule et al. [99] conducted group discussions and on-site observations of poultry farms in Ghana to assess biosecurity practices. El Masry et al. [106] employed transect walks, household bird observations, and key informant meetings to verify findings and identify gaps. Kang et al. [90] targeted key locations within poultry markets, such as cages, waste bins, and slaughter areas, to conduct environmental swab sampling and identify contamination hotspots. Kwan et al. [112] validated survey responses through field observations at ports, revealing discrepancies in reported contact with dogs. Lauterbach et al. [95] observed hand sanitation practices at agricultural fairs, measuring intervention uptake alongside ongoing disease surveillance efforts. These findings demonstrate how community-centered data collection that integrates multiple methods and levels of engagement produces more diverse datasets that are essential for understanding and preventing spillover events.

## 4. Discussion

Fifty-five out of the ninety-five articles analyzed did not have any level of community engagement reported, and no articles had evidence of community-driven or community-led decision-making. For those articles that had some level of community engagement, relevant elements included the platforming of community consultation, community training for involvement in spillover prevention research, the engagement of cultural and language considerations, community protection and awareness programmes for public health and biosecurity, and community-centered data collection processes. Our findings highlight substantial gaps in the kinds of community engagement activities used within the evaluation of public policies for spillover prevention. Furthermore, the breadth of the findings and the types of policies reviewed point to a likely high level of low to no community engagement across all stages of public policy design, research, and implementation.

Community engagement, as it is most often practiced, has often been transactional in nature (i.e., minimal level of relations, time-bound) in policy and practice settings [127]. In certain circumstances, community engagement has even been performative or tokenistic, aligning with an arguable checkbox-like approach [128]. Given this, relevant or affected communities are often engaged only after programmes or evaluation protocols have been established, key decisions made, and leadership appointed, or they are consulted in a way that lacks decision-making or governance power [129]. Even with more participatory research and evaluation approaches, power imbalances still often remain, depending on the purpose of the work, which dictate how “power dynamics, including inequities, biases, discrimination, racism, rank and privilege, are handled within the collaborative arrangement” [130].

Community engagement, both as a process and a practice, is also influenced by the context in which it is implemented. Given the dominance of Euro–Western-centric worldviews, along with the methodologies, methods, and policy models [131] that often accompany them, community engagement tends to be shaped within this Euro–Western worldview framework. As a result, the approaches to community engagement may unintentionally reflect the hierarchical structures embedded in Euro–Western research, policy, and practice environments. These hierarchical structures frequently marginalize alternative ways of knowing (such as Indigenous knowledge), prioritize Euro–Western education over lived experience, devalue cultural and language diversity, and typically retain final decision-making authority within the team leading the community engagement process. Our analysis found that only five out of ninety-five articles scored a 3 (indicating work was carried out directly with the community) or a 4 (indicating the community was a direct partner in the process), and none scored a 5 (indicating community-led decision-making). This highlights the continued prevalence of “information giving” and “consultative” approaches, which fail to address existing power imbalances and limit the effectiveness of community engagement strategies in producing meaningful outcomes [132].

Given that many spillover prevention activities are at the nexus of various communities (e.g., farmers, Indigenous Peoples, pastoralists, food-related vendors, etc.) [133], top-level decision-making without the direct involvement of communities will continue to challenge intervention success, making it more difficult to build effective relationships for lasting impact [134,135]. Relational community engagement (see Box 1) has been increasingly platformed as a genuine approach to co-building partnerships. This includes understanding the importance of co-design, co-production, co-learning, and co-evaluation processes; understanding engagement as an ongoing process that requires time, commitment, and resources for all sides; and recognizing that communities are made up of diverse populations, which may require a unique approach within a broader community engagement approach [26]. With this, there have been calls from within WHO-related work to “develop consensus on adopting a relationship-focused approach to community engagement as an inherent way of working in health systems and across sectors”, and to “strengthen foundational engagement competencies, skill sets and processes within and across health services” [26]. Relational community engagement interventions have been shown to result in “increased trust between stakeholders and groups/teams, and increased community senses of ownership of interventions, decisions, structures”, as well as having the potential to influence broader societal factors and having “positive impacts on health policy and governance including collaboration between sectors and communities as well as increased access to services” [134].

Box 1. **Definition and tenets of relational community engagement.**

- Relational community engagement is an approach that conjoins individual and collective awareness and is intentional about processes that facilitate positive connection, belonging, and communication—all of which are needed for meaningful collaboration and co-production [134].
- This approach emphasizes and centers on building nurturing, ongoing, and longstanding relationships between different stakeholders, including community members, organizations, and institutions for improved health outcomes [134].
- Rather than focusing solely on specific projects or outcomes, relational community engagement places a strong emphasis on developing and maintaining long-term connections and trust within communities [134].

Platforming holistic and community-centric participatory approaches to address diverse health and social challenges adds potential versatility and effectiveness to interventions that rely on effective relationships [134]. Given the complexity of zoonotic spillover prevention, relational community engagement could be considered as foundational for any intervention, policy, or otherwise. Furthermore, relational community engagement could be seen as the intervention itself, setting the context for solutions to emerge. Regardless, spillover prevention work does not need to reinvent the wheel; however, the work will require resetting the priorities towards meaningful community engagement and more balanced decision-making power to address equity issues, with the potential for strong co-development and partnership engagements to occur within settings of increased cultural diversity.

### Limitations

Since our secondary analysis for this study was based on the search criteria of the original systematic review, it is limited by the comprehensiveness of that review. The original review acknowledged the potential for relevant policy evaluations to be overlooked due to the complex drivers of spillover events, as well as its focus on “peer-reviewed literature and the grey literature published by international agencies and organizations.” As a result, policies that have been implemented but not evaluated, or evaluated but not published in this literature body, may have been missed [25]. Regardless, as our secondary analysis was intended to gain a better appreciation of the levels of community engagement being used as well as the kinds of community engagement elements being utilized, we feel our results demonstrated a clear trend of gaps across the published literature that needs further highlighting. Our secondary analysis also relied on the articles that explicitly described any relevant community engagement activities carried out. It is possible that some articles engaged in more community engagement activities than were described in the published papers; however, this cannot be confirmed. This issue underscores the need for more consistent standards in reporting the levels of community engagement used in projects to ensure greater transparency. We are concerned, however, that the lack of described community engagement strategies may reflect limited or no community engagement being carried out across those studies. Lastly, we did not apply any metric or measurement of community engagement policy success in the classification of community engagement, which was outside the scope of our current study. Regardless, subsequent studies would benefit from moving toward an evaluative focus on effectiveness for community engagement approaches.

## 5. Conclusions

Our findings highlight that there continues to be significant gaps in appropriately engaging affected communities within the research, evaluation, and implementation of public policies for spillover prevention. With this, further effort is needed to ensure equity and community-centered approaches are platformed explicitly in future primary spillover prevention activities. The implementation of effective spillover prevention policies will be dependent on several factors, including the meaningful engagement of relevant stakeholders, the ability to anticipate potential resistance to any policy intervention, and ensuring flexibility to adapt to changing circumstances. Understanding these factors necessitates the platforming of relational engagement approaches that make space for local expertise, including diverse knowledges, while also considering local contexts, cultures, and community-level decision-making, and fostering trust. We call for greater platforming of relational community engagement approaches as well as making meaningful space for community-based leadership within spillover prevention research, policy, and practice.

## Figures and Tables

**Table 1:** Matrix of analysis for the level of community engagement within the included research papers (adapted from Vaughn & Jacquez [28] and Key et al. [29]).

No Community Involvement Noted	Community Informed: Information Is Provided to Community	Community Consultation: Input Is Obtained from Community	Community Involvement: The Work Is Carried out Directly with Community	CBPR */Community Collaborate: Community Is Partner in the Process	Community Driven/Led: Community Leads Decision Making
0	1	2	3	4	5

* CBPR: Community-based participatory research.

**Table 2:** Distribution of community engagement scores across the evaluated studies.

Score	Frequency n (%)	Articles
0	55 (57.9)	Abbas et al. [32], Anderson et al. [33], Backer et al. [34], Backer et al. [35], Basinski et al. [36], Berry et al. [37], Beyer et al. [38], Brennan et al. [39], Busani et al. [40], Cardador et al. [41], Chen et al. [42], Chowell et al. [43], Cuthbert et al. [44], Davis et al. [45], De Lucca et al. [46], Fournié et al. [47], García-Díaz et al. [48], Gordon et al. [49], Graiver et al. [50], Häsler et al. [51], Hegazy et al. [52], Huot et al. [53], Kangas et al. [54], Kung et al. [55], Liu et al. [56], Lu et al. [57], HuaKun et al. [58], Ma et al. [59], Mendez et al. [60], Mroz et al. [61], Naletoski et al. [62], Okello et al. [63], Pinsent et al. [64], Rasouli et al. [65], Roy et al. [66], Sanchez et al. [67], Selhorst et al. [68], Shwiff et al. [69], Shwiff et al. [70], Smith et al. [71], Teng et al. [72], Todd Weaver et al. [73], Tustin et al. [74], Walker et al. [75], Wang et al. [76], Wang et al. [77], Weaver et al. [78], World Health Organization [79], Wilson et al. [80], Wu et al. [81], Wu et al. [82], Xing et al. [83], Yu et al. [84], Yuan et al. [85], and Zhu et al. [86]
1	12 (12.6)	Andronico et al. [87], Hassim et al. [88], Horigan et al. [89], Kang et al. [90], Karabozhilova et al. [91], Karki et al. [92], Kimani et al. [93], Knight-Jones et al. [94], Lauterbach et al. [95], Lewis et al. [96], Li et al. [97], and Vivancos et al. [98]
2	23 (24.2)	Akunzule et al. [99], Amparo et al. [100], Bonwitt et al. [101], Brinkley et al. [102], Brooks-Moizer et al. [103], Campbell et al. [104], De Serres et al. [105], El Masry et al. [106], Ferguson et al. [107], Fournié et al. [108], Guerrier et al. [109], Huang et al. [110], Kung et al. [111], Kwan et al. [112], Lin et al. [113], Manyweathers et al. [114], Manyweathers et al. [115], Oladokun et al. [116], Roth et al. [117], Stewart et al. [118], Swayne et al. [119], Turkson et al. [120], and Yee et al. [121]
3	3 (3.1)	Bechir et al. [122], Hunter et al. [123], and Ministry of Agriculture [124]
4	2 (2.1)	Samaan et al. [125], and Thomas et al. [126]
5	0 (0.00)	-

**Table 3:** Themes and definitions.

Themes	Descriptions
Platformed community consultation in spillover prevention research	Studies incorporated processes for seeking insights, specialized knowledge, and feedback from community experts and stakeholders to inform the development, refinement, and validation of research methods and health interventions. This form of engagement better ensured the studies were culturally relevant and aligned with community needs.
Community training for involvement in spillover prevention research	There were efforts to actively involve community members and local stakeholders by providing education and training that equipped them to participate effectively in the planning, implementation, and evaluation phases of health interventions and research activities.
Cultural and language considerations engaged within spillover prevention research activities	There were efforts to honour and accommodate cultural diversity in research by using appropriate language and translators, while tailoring communication and data collection tools to reflect the cultural and linguistic preferences of community members.
Community protection and awareness programmes for public health and biosecurity	There were efforts to implement integrated initiatives that combined education, awareness-raising, and protective measures to strengthen biosecurity and build community resilience against disease transmission.
Community-centerd data collection for spillover prevention research	Community members were engaged as active participants in data collection, recruitment, and consent processes. In addition, diverse methods were employed to generate datasets for understanding and mitigating spillover risks.

## Data Availability

The original contributions presented in this study are included in this article. Further inquiries can be directed to the corresponding authors.

## Abbreviations

The following abbreviations are used in this manuscript:

CE	Community engagement
WHO	World Health Organization
